# Persistence of pharmacological migraine prevention based on statutory health insurance claims data from Germany: a retrospective cohort study

**DOI:** 10.3389/frhs.2026.1934185

**Published:** 2026-10-29

**Authors:** Andreas Domdey, Sonja Gscheidle, Heiko Löhr, Patrizia Maria Maier, Matthias Arnold

**Affiliations:** 1 Lundbeck GmbH, Hamburg, Germany; 2 inav Institute for Applied Health Services Research (inav GmbH), Berlin, Germany

**Keywords:** aCGRP-mAbs, Clostridium botulinum neurotoxin type a, health claims data, Kaplan–Meier survival curves, migraine prevention, monoclonal antibodies, persistence, real-world-evidence

## Abstract

**Introduction:**

In the global population aged 20–99 years, migraine shows the second greatest loss of disability-adjusted life years (DALYs). In Germany, migraine affects around 15% of all women and 6% of all men. Migraine preventive therapy aims to reduce the frequency, severity and duration of attacks. We evaluated the persistence of conventional drug therapies for migraine prevention under real-world conditions focusing on defined oral drugs and Clostridium botulinum neurotoxin type A (BoNTA) in patients who were subsequently prescribed aCGRP monoclonal antibodies.

**Methods:**

Claims data from German statutory health insurance were used to analyze prescription patterns. Prevalent migraine patients were defined as individuals over 18 years of age at the beginning of the observation period with at least one ICD-10 G43 diagnosis. Persistence of oral prophylactic therapies and BoNTA was determined retrospectively in migraine patients with a first prescription of an aCGRP-mAb between October 1 and December 31, 2021 and uninterrupted retrospective data over the preceding five years. Persistence was defined as 180 days of uninterrupted preventive therapy with the same as the first prescription. Prescription duration was based on the therapeutic range per prescribed package according to the prescribing information. Kaplan–Meier survival analyses were conducted to evaluate the time until treatment discontinuation.

**Results:**

For the analysis of conventional therapies, a cohort of 137 patients was identified. The 137 patients (119 females and 18 males) received an average of 2.6 conventional preventive therapies during the five-year observation period before the first monoclonal antibody prescription. For most of the preventive drugs less than five patients fulfilled the persistence criteria on 180 days. On average, patients began therapy with a monoclonal antibody more than one year after the last previous conventional therapy was dispensed.

**Discussion:**

The persistence of oral preventive migraine treatments not specifically developed for migraine prevention was low, suggesting either temporary relief from migraine or treatment discontinuation for reasons that could not be identified from the data used in this study. Further systematic investigations comparing the persistence of conventional preventive therapies and aCGRP-mAbs in routine clinical practice in Germany are warranted.

## 1 Introduction

In the global population between 20 and 99 years of age, migraine shows the second greatest loss of disability-adjusted life years (DALYs; 750.8 per 100,000 people), according to the Global Burden of Disease Study (2021) (1)

Migraine ranks second as cause of years living with disability (YLD) cause among patients aged 15 to 49 years (2). Migraine is the main cause of life years lost and the second most common reason for disability in general; in women it is the most common reason for disability (3). In Germany, similar to other countries, migraine affects around 15% of all women and 6% of all men (4). According to the ICHD-3 diagnostic criteria, a distinction is made between episodic (EM) and chronic (CM) migraine.^1^ In most patients (approximately 92%–94%) (5–7), migraine is categorized as “episodic”.

Acute migraine attacks can be treated with various pharmacological modalities including non-steroidal anti-inflammatory drugs (NSAIDs) and triptans. However, acute therapy is often not sufficient to reduce the burden of migraine patients, and overuse of triptans and opioids has been shown to increase the frequency of headaches (8). Reasons include insufficient effectiveness of the medications (9), occurrence of adverse events, safety concerns that prevent prescription of medications (10), or occurrence of medication overuse headache (MOH), a secondary headache resulting from excessive use of analgesics or triptans (11).

To mitigate these limitations, migraine prevention is recommended for patients with high disease burden. The goal of preventive treatment of migraine is to reduce the frequency, intensity, and duration of attacks. In the case of episodic migraine, successful treatment is defined as a reduction in the average monthly migraine days by 50% or more, in the case of chronic migraine of 30% or more (12, 13).

In Germany, approved pharmacological treatments for migraine prevention include beta-blockers (metoprolol, propranolol), amitriptyline, flunarizine, topiramate and *Clostridium botulinum* neurotoxin type A (BoNTA, only for chronic migraine) (14). These agents are considered first-line preventive treatments, each with distinct modes of action, tolerability profiles and contraindications (12). Valproic acid could be used off-label for migraine prevention if treatment with other drugs approved for migraine prevention was unsuccessful, however this treatment option for migraine prevention is strongly limited since early 2020 (15). Oral migraine preventive medications are often associated with tolerability issues and discontinuation rates are relatively high, with more than 80% of patients discontinuing treatment within the first year (16, 17).

Since 2018, monoclonal antibodies specifically targeting migraine pathophysiology via the calcitonin gene-related (CGRP) peptide pathway (18) have been approved and marketed in Germany: erenumab became the first monoclonal antibody to receive European approval for migraine prevention, followed by galcanezumab and fremanezumab in 2019, as well as eptinezumab in 2021 (19). These new agents have demonstrated efficacy and favorable tolerability across various studies and patient populations (12).

The introduction of these monoclonal antibodies targeting CGRP or its receptor (aCGRP-mAbs) represented a significant advance in migraine prevention in clinical practice; however, their high costs remain a barrier to broad use (10), and guidelines required to use the approved conventional pharmacological treatments for migraine prevention as first-line treatments before switching to an aCGRP-mAb in clinical practice (12).

There is currently limited real-world data from statutory health insurance (SHI) companies that has been analyzed on prevalence, burden of disease, usage and persistence of migraine preventive medications in Germany (20–23). Analyses of healthcare data from other countries suggest an association between decreased persistence and increased utilization of healthcare resources in migraine treatment (24, 25).

The aim of this study was to use data from the statutory health insurance system (SHI) to evaluate the number and duration of conventional preventive oral therapies and treatment with BoNTA for migraine prevention under real-world conditions in Germany. In addition, the time period between the last conventional treatment and the first prescription of an aCGRP-mAb was to determined.

## 2 Materials and methods

### 2.1 Study design

This was an observational retrospective cohort study of German SHI claims data to analyze the persistence of treatment with beta-blockers (metoprolol, propranolol), amitriptyline, flunarizine, topiramate, valproic acid and BoNTA as recommended first-line treatments for migraine prevention. The study period ran from January 1, 2016 to December 31, 2021 and was divided into a retrospective observation period and an index period starting on October 1, 2021. Patients were indexed based on the date of their first aCGRP-mAb prescription for migraine prevention between October 1, 2021, and December 31, 2021 (index period). Identified patients were retrospectively followed without interruption, starting exactly five years before their index date. All patients had to be fully observable during this period.

aCGRP-mAbs included erenumab, fremanezumab and galcanezumab. Eptinezumab was not available in Germany at the defined index period. Drugs analyzed during the retrospective observation period included beta-blockers (metoprolol, propranolol), amitriptyline, flunarizine, topiramate and BoNTA.

### 2.2 Data source

Data for this study were derived from the InGef Research Database, which contains anonymized billing data from approximately 8,8 million insured individuals in Germany. This database has been shown to be representative with respect to morbidity, mortality, and healthcare utilization for the entire population of 74 million SHI-insured individuals in Germany (26). Claims data according to § 300ff of the German Social Code Book V (SGB V) were transmitted as social data for research and planning purposes pursuant to § 75 German Social Code Book X (SGB X). Under this legal framework, the data are made available in anonymized form for scientific research purposes. As the analyses are based exclusively on anonymized secondary data provided in accordance with statutory regulations, informed consent from the insured individuals was not required.

### 2.3 Inclusion and exclusion criteria

The cohort consisted of patients with their first prescription claim for erenumab, fremanezumab, or galcanezumab between October 1, 2021, and December 31, 2021 which were the only aCGRP options available in Germany at the time. Patients had to have at least one documented ICD-10 G43 diagnosis during the entire observation period, according to ICD 10-GM version 2021) (27). The search for G43 diagnoses encompassed G43.-, G43.0, G43.1, G43.2, G43.3, G43.8, and G43.9. In addition, patients were required to be 18 years or older at the start of the observation period. Patients were excluded if they did not have continuous insurance coverage or if their medication history could not be fully traced without interruption throughout the whole study period, or if they had been prescribed an aCGRP mAb prior to the index period (Figure 1).

**Figure 1:**
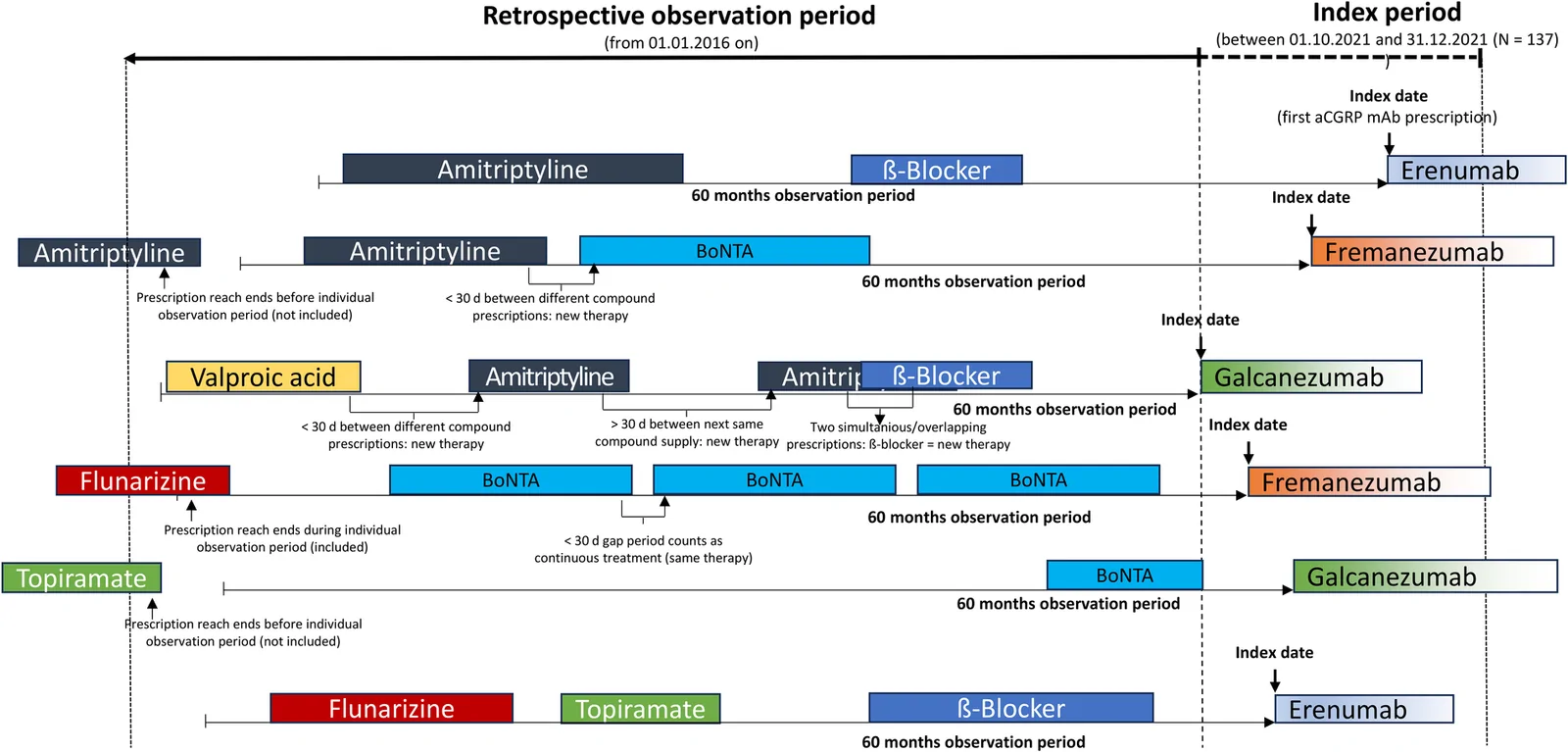
Illustration of different schematic patient cases and their respective observation periods.

### 2.4 Persistence measurement

The analysis was based on a retrospective treatment episode analysis. A treatment episode was defined as an uninterrupted treatment sequence with the same preventive drug, allowing a maximum permissible gap of 30 days (gap period) between the calculated end of prescription coverage and the subsequent refill. during which the patient receives a new supply of no more than 30 days (gap period) after termination of the days’ supply of the preceding treatment. Each patient was followed up retrospectively for exactly five years starting with their respective index date.

Persistence (28) was defined as ≥ 180 days of uninterrupted treatment sequence with the same preventive drug starting with the initial prescription, allowing a maximum permissible gap of 30 days between the calculated end of each prescription coverage and the subsequent refill (29, 30). Patients who remained persistent beyond 180 days were considered persistent at the 6-month landmark.

The duration of 180 days was established as a compromise between long-term preventive treatment goals, the possibility of taking into account two courses of BoNTA in accordance with the summary of product characteristics (31), and recommendations to discontinue treatment with flunarizine after six months (32). To reflect also later-stage persistence, we used a duration of 180 days to accommodate two refills (29). Switching was defined as initiation of a different migraine preventive drug without subsequent continuation of the index drug within the 30-day permissible gap. Concomitant or overlapping prescriptions of different preventive drugs were not considered a switch as long as the index drug continued according to the defined episode logic. Prescription coverage periods were determined for each patient based on defined daily doses (DDDs) according to ATC classification codes and dosing recommendations specified in the respective prescribing information (Table 1). In the case of BoNTA all formulations approved for the prophylactic treatment of chronic migraine were considered. DDDs or Allergan units prescribed in the same quarter in which one of the migraine diagnoses was made were calculated for each package according to the prescribing information (identified by their German pharmaceutical registration number). An episode ended when no refill of the same active ingredient occurred within 30 days after exhaustion of the previous supply or when treatment with the same drug was permanently discontinued.

**Table 1:** Included preventive drugs for migraine prevention analyzed in this study.

Preventive drug	Class	ATC^a^ (50, 51)	Dose
Erenumab	CGRP-Receptor antagonist	N02DC01/ N02CX07	70 mg and 140 mg
Galcanezumab	CGRP-Ligand antagonist	N02DC02/ N02CX08	120 mg
Fremanezumab	CGRP-Ligand antagonist	N02DC03/ N02CX09	225 mg
			
BoNTA	Neuromuscular blocking agent	M03AX21^b^	50–200 Allergan units
Amitriptyline	Antidepressants	N06AA09	50–75 mg
Flunarizine	Calcium channel blocker	N07CA03	5–10 mg
Metoprolol	Beta blocker	C07AB02	50–200 mg
Propranolol	Beta blocker	C07AA05	40–240 mg
Topiramate	Anticonvulsant	N02CX12/ N03AX11	25–100 mg
Valproic acid	Anticonvulsant	N03AG01	500–1,000 mg

a Between 2019 and 2020, a change in ATC codes for erenumab, fremanezumab, and galcanezumab took place.

b Only local identifiers (“PZN”) with approval for prophylactic treatment of chronic migraine were included.

During the observational period, patients could receive prescriptions for multiple active ingredients either simultaneously, sequentially, or with overlapping treatment episodes. Each prescription with the same active ingredient after a gap period or overlap with another prescription was counted as additional therapy. Mean duration of conventional migraine preventive therapies was calculated based on the number of patients with coverage ranges for the respective medication.

Oral therapies were included even if they were prescribed for a non-migraine indication, as their potential effectiveness for migraine was assumed. Since all patients had at least one migraine diagnosis at the time of the respective aCGRP-mAb prescription, it was considered appropriate to expect possible migraine prevention effects caused by these oral therapies. Information on all product forms was obtained from Lauer-Taxe®, a database of all pharmaceutical entities, including drugs, medical devices, and their prices (33).

### 2.5 Outcomes variables

The primary outcome was 180-day-persistence to the following migraine preventive drugs: beta blockers (propranolol and metoprolol), flunarizine, topiramate, valproic acid, amitriptyline and BoNTA reported at day 180 after treatment initiation.

Secondary outcomes were the time to treatment discontinuation for each preventive drug, the number of different preventive drug therapies per patient, mean treatment duration for each preventive drug and the average time interval between the end of the last conventional preventive treatment and the initiation of an aCGRP-mAb therapy (index date). This interval was determined for each patient based on the dispensing date of the last non-aCGRP-mAb prophylactic therapy and the first dispensing date of an aCGRP-mAb prescription.

### 2.6 Statistical analysis

All analyses conducted were descriptive. No formal sample size calculation was carried out for this study given the retrospective and descriptive nature of the analysis. Except for gender, no covariates were defined *a priori* due to real world nature of the data source, which did not provide data on migraine severity, region or type of prescribing physician.

The time to treatment discontinuation was analyzed using Kaplan–Meier (KM) survival curves separately for each preventive drug prescribed during the retrospective observation period with right censoring of patients who received a continued prescription of the drug after the end of the index period (i.e., after December 31, 2021). KM curves were estimated using a stricter discontinuation definition that did not consider the 30-day gap period; in this analysis, discontinuation was defined as the absence of a refill immediately following the calculated prescription coverage period. Different preventive drugs prescribed subsequently, simultaneously, or overlapping for one patient were considered each as single therapy in the respective KM-curve.

The mean treatment duration for each preventive drug was calculated based on the observed treatment durations (i.e., not derived from KM estimates). In cases of right censoring, treatment duration was calculated up to December 31, 2021.

## 3 Results

A total of 137 patients were identified who received a prescription for an aCGRP-mAb for migraine prevention for the first time between October 1 and December 31, 2021, and whose preventive migraine medications were retrospectively monitored over the preceding five years observation period. Among these patients, 87% (*N* = 119) were females and 13% (*N* = 18) were males. On average, patients received 2.6 preventive treatments during the five-year observation period prior to initiation of aCGRP-mAb therapy. 44% of the observed patients had undergone zero to two, no one more than 5 previous therapies with conventional prophylactic agents during the study period. In total, 356 prior therapies were identified, of which 301 were administered to female patients and 55 to male patients (Table 2).

**Table 2:** Number of conventional therapies for migraine prevention in patients with an aCGRP-mAb initiation.

Patients characteristics	N total (%)	Female, N (%)	Male, N (%)
All patients	137 (100)	119 (87)	18 (13)
without conventional therapies	19 (14)	18 (15)	1 (1)
with 1 conventional therapy	13 (10)	12 (10)	1 (1)
with 2 conventional therapies	28 (20)	24 (20)	4 (3)
with 3 conventional therapies	29 (21)	26 (22)	3 (2)
with 4 conventional therapies	40 (29)	32 (27)	8 (45)
with 5 conventional therapies	8 (6)	7 (6)	1 (1)
Total conventional therapies	356	301	55
Total conventional therapies/patient (mean)	2.6	2.5	3.1

After 180 days of treatment with conventional oral therapies, the number of persistent patients was fewer than five for most of the preventive drugs (Table 3). Exceptions were amitriptyline and BoNTA, with 6.6% and 100% 180-day persistence, respectively.

**Table 3:** Number and proportion of persistent patients at day 1 and 180 in previous therapies by active ingredient/ATC classification.

	Patients with conventional preventive therapy, N (%)			Persistent patients with 180 days continuous prescription of the same preventive drug (including gap period), N (%)		
Preventive drug	Total^a^ (*N* = 137)	Female^a^ (*N* = 119)	Male^a^ (*N* = 18)	Total	Female	Male
BoNTA (M03AX21)	30 (22)	25 (21)	5 (28)	30 (100)	25 (100)	5 (100)
Amitriptyline (N06AA09)	94 (69)	80 (67)	14 (78)	7 (7)	<5^b^	<5^b^
Flunarizine (N07CA03)	54 (39)	43 (36)	11 (61)	<5^b^	<5^b^	0
Metoprolol (C07AB02)	55 (40)	45 (38)	10 (55)	<5^b^	<5^b^	0
Propranolol (C07AA05)	16 (12)	14 (12)	2 (11)	0	0	0
Topiramate (N03AX11, N02CX12)	100 (73)	89 (74)	11 (61)	<5^b^	0	<5^b^
Valproate (N03AG01)	7 (5)	5 (4)	2 (11)	0	<5^b^	0

a Patients prescribed more than one preventive drug were counted multiple times.

b Results based on 1–4 patients were masked for patient privacy.

The average duration of the respective therapies per patient, regardless of the preventive medication, was usually less than 2.5 months, and approximately 2.5 years for BoNTA (Table 4).

**Table 4:** Mean duration of individual conventional migraine preventive therapies (in days).

Preventive drug	All patients^a^ (*N* = 137)	Female^a^ (*N* = 119)	Male^a^ (*N* = 18)
BoNTA (M03AX21)	935	955	834
Amitriptyline (N06AA09)	56	61	24
Flunarizine (N07CA03)	69	76	45
Metoprolol (C07AB02)	30	31	27
Propranolol (C07AA05)	13	15	0
Topiramate (N03AX11, N02CX12)	11	9	30
Valproate (N03AG01)	30	36	15

a Patients with at least one prescription during the observation period. Values are rounded to integers. The mean treatment duration for each preventive drug was calculated based on observed treatment durations (i. e., not derived from KM estimates). In cases of right censoring, treatment duration was calculated up to December 31, 2021.

Figure 2 illustrates therapy discontinuation for conventional migraine preventive therapies in the defined population. KM survival curves represent the cumulative supply range of the prescribed formulations for each preventive drug. A steeper decline indicates lower mean persistence to the respective preventive drug. Confidence bands (95% confidence intervals) are shaded in gray. For BoNTA the difference between persistence (Table 3) and KM survival curves of two survivors on day 180 is related to the 30-day gap period that has been considered only in the persistence definition to reflect real-world prescription patterns (Supplementary Table S1).

**Figure 2:**
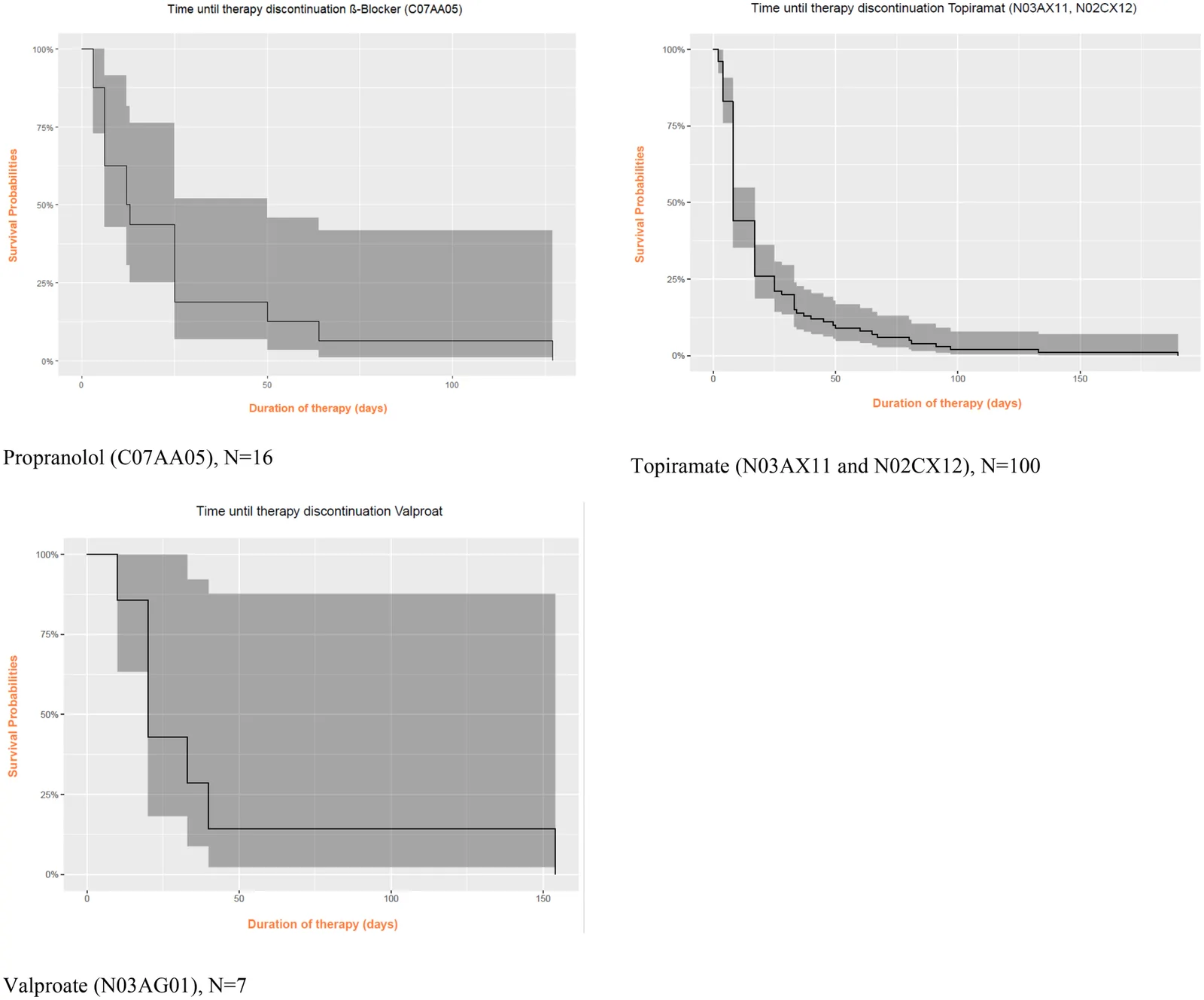

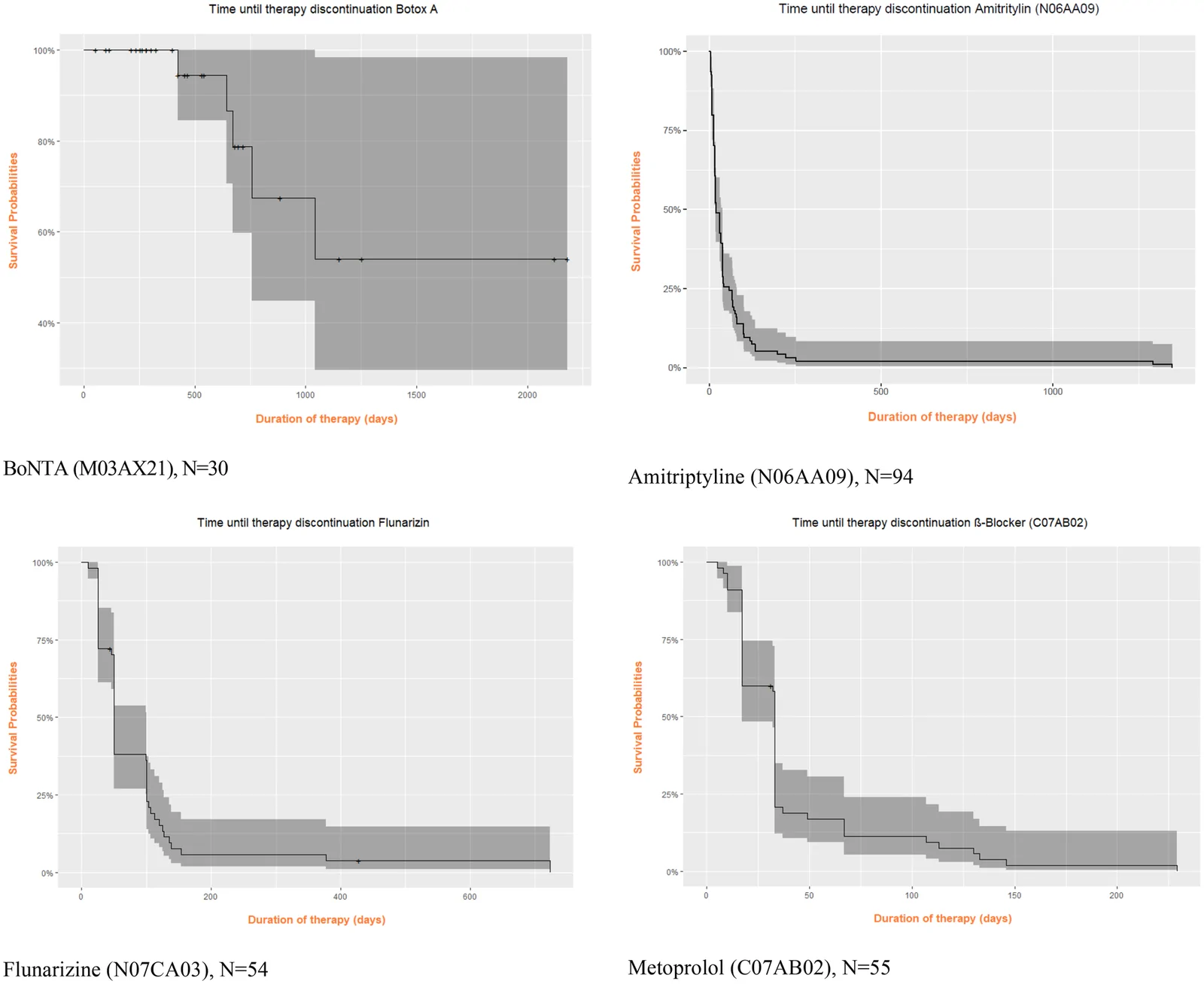
Time until therapy discontinuation by preventive drug (+ indicates censored observations).

Of all 137 patients, 78 switched from conventional therapies to an aCGRP-mAb during their follow-up period. Forty patients continued to receive conventional migraine preventive medication after the initial initiation of aCGRP-mAb therapy, while 19 patients had not previously received any therapy with conventional therapy during the retrospective observation period. The time between the last conventional therapy (date of dispensing) and the start of aCGRP-mAb was 368 for all patients (364 days for female, 392 for male; Table 5).

**Table 5:** Switching characteristics and mean duration until initiation of an aCGRP-mAb.

Patients characteristics	N total (%)	Female, N (%)	Male, N (%)
All patients	137 (100)	119 (87)	18 (13)
Patients who switched from conventional therapy to an aCGRP-mAb	78 (57)	66 (55)	12 (67)
Patients without conventional therapies	19^b^ (14)	18^b^ (15)	1^b^ (1)
Patients with conventional therapies dispensed after aCGRP-mAb initiation	40 (29)	35 (29)	5 (28)
Mean duration until first aCGRP-mAb prescription in days (SD)^a^	368 (562)	364 (573)	392 (513)

a dispensation date was considered. Values are rounded to integers.

b all before 2020.

## 4 Discussion

In this study, we have analyzed the persistence of conventional drug therapies for migraine prevention who were subsequently prescribed an aCGRP-mAb therapy. Data were derived from a SHI prescription claims database and therefore indicative for real-world conditions in Germany.

Our data adds further evidence to existing studies showing that a majority of migraine patients exhibit low persistence to conventional oral therapies before initiating an aCGRP-mAb treatment for migraine prevention. The calculated high discontinuation rates within the first 180 days for oral regimes may be caused by insufficient effectiveness, intolerable side effects, or poor adherence due to missed or forgotten doses. In contrast, persistence was markedly higher in patients receiving BoNTA for chronic migraine, with 100% persistence at 180 days (persistence definition) or 93% in the KM-Analysis, in which the 30-day gap definition is not considered. Due to the retrospective study design and the use of anonymized data, no influence on prescribing behavior was exerted.

Based on the outcomes of this analysis it can be assumed that most patients initiating an aCGRP-mAb could not be treated adequately with conventional oral therapeutics for prevention. Supported by the KM survival curves and consistent with similar studies (34, 35) our data instead suggest that sustained preventive migraine therapy with oral preventive therapeutics over five years was either unsatisfactory or inadequate for most patients and/or treating physicians within this patient population.

Most patients in this study were female, many of whom were of childbearing age, which restricted the availability of certain therapeutic options. The observed ratio of 87% female to 13% male is not fully consistent with reports on migraine prevalence in Germany of around 15% of all women and 6% of all men (4), but aligns more closely with results of gender related research on migraine prevalence (e.g., 32.9% for women, 13.0% for men) (36) and migraine recognition rates (82% women, 18% men) (37).

From an economic perspective, an average of 2.6 oral preventive therapies per patient appears reasonable, as each therapy initiation may increase the likelihood of identifying an individually effective and efficient treatment. However, arguments against this approach include potential adverse events, contraindications associated with these active ingredients and avoidable continued migraine episodes during and after periods of insufficient preventive treatment.

Substance-specific assessments have been published by the European Headache Federation (EHF) in 2023 and 2024, based on current evidence for topiramate, flunarizine, amitriptyline, and propranolol. According to the EHF, high-quality evidence supports the efficacy of topiramate in migraine prophylaxis. However, the risk of adverse events that may lead to treatment discontinuation is regarded as a major concern (38). For flunarizine, existing evidence does not align with the recommended endpoints for evaluating migraine prophylaxis medications, making adequate assessment impossible. The EHF therefore advocates for modern, large-scale studies to reassess the efficacy of flunarizine for migraine treatment (39). Additionally, according to the prescribing information, flunarizine should not be used for longer than six months (32), limiting its suitability for long-term migraine prevention. Regarding amitriptyline, the EHF criticized the lack of robustness of the available study results (40).

Numerous publications confirm the efficacy of beta-blockers for migraine prevention, particularly metoprolol and propranolol, and recognize the quality of studies on these two substances (41–44). The EHF highlights the prophylactic role of propranolol in migraine with an overall acceptable tolerability profile and confirms its role as a first-line agent in the prophylaxis of migraine (41). However, evidence for the longer-term effectiveness and persistence of beta-blockers in migraine prevention in real-world clinical practice is lacking (42–44), described as poor or has not been adequately assessed (45, 46).

German guidelines and the requirements of statutory health insurance funds changed in mid-to-late 2019 and then required four (or five in the case of chronic migraine) conventional preventive therapies before an aCGRP-mAb for migraine prevention can be prescribed (47–49). Analysis shows that all initial prescription of an aCGRP-mAb without any previous therapies happened from July 1 to December 31, 2019, indicating that physicians later reacted rapidly to the new recommendations. Later, in 2020/2021, the eligibility of valproic acid for off-label prescription for migraine prophylaxis was effectively revoked (15), as its use was contraindicated in women of childbearing age (12). As a result, valproic acid currently no longer plays a role in migraine prophylaxis in Germany.

Although oral preventive drugs recommended for migraine prevention in Germany demonstrate effectiveness, their long-term therapeutic value must be viewed critically for some patients because of their higher risk of adverse events, or contraindications (25). None of these drugs were specifically developed for migraine prevention. These factors contribute to a decline in persistence for all active ingredients, except for BoNTA.

The mean interval between the discontinuation of the last previous therapy and the initiation of an aCGRP-mAb treatment was approximately one year per patient. This timeframe may be slightly overestimated, as the analysis was based on the dispensing date of the last prior therapy, assuming the medication was subsequently taken as prescribed. Patients who continued to take conventional migraine preventive medication after starting aCGRP-mAb therapy may have needed these medications for other purposes, e.g., for high blood pressure or psychiatric disorders.

### 4.1 Limitations

The observation period for assessing prescription of migraine preventive therapies was five years. Consequently, it must be assumed that patients were not included in the study population because they may have already received oral prophylactic treatment before the start of the observation period or were unable to receive such treatment due to contraindications. Small numbers of patients taking certain oral preventive drugs (e.g., valproic acid) may limit the significance of persistence under the given definition. To clarify this issue, a matched-pair analysis with a larger patient cohort would be necessary.

Data from German SHI companies collected under § 300 ff. SGB V and § 75 SGB X does not include information on monthly headache or migraine days. Therefore, the persistence data cannot be correlated with clinical outcomes such as the effectiveness of the prescribed preventive drugs or with changes in monthly migraine days with or without preventive migraine medication. As a result, this study could not examine whether monoclonal antibodies were primarily prescribed to patients who did not respond to conventional oral therapies, or whether the low persistence of conventional oral treatments represents a class effect affecting most patients undergoing migraine prophylaxis. Given the one-year gap (on average) between the last conventional prescription and the first prescription of an aCGRP-mAb, and considering the retrospective nature of the analysis, it is assumed that the persistence results can also be generalized to patients who did not ultimately receive an aCGRP-mAb therapy.

## 5 Conclusion

In a majority of migraine patients, the initiation of aCGRP-mAb preventive treatment follows a period of ineffective treatment with conventional oral preventive treatment. In this group, the persistence of oral preventive migraine treatments not specifically developed for migraine prevention was low, suggesting either temporary relief from migraine or therapy discontinuation for reasons that could not be identified from the data used in this study (e.g., non-sustained therapeutic effectiveness or side effects). The following initiation with aCGRP is indicative for the need of longer-lasting effective treatment alternatives for migraine patients. Further systematic investigations comparing the persistence of oral preventive therapies and aCGRP-mAbs in preventive treatment-naïve migraine patients in Germany are warranted.

## Data Availability

The data analyzed in this study is subject to the following licenses/restrictions: The data are provided by InGef GmbH and are not publicly available. Requests to access these datasets should be directed to info@ingef.de.

## Footnotes

^1^Episodic migraine according to IHS: Less than 15 headache days per month
