# Supplementary material for: Persistence of pharmacological migraine prevention based on statutory health insurance claims data from Germany: a retrospective cohort study
Source: Front Health Serv. 2026 Oct 29;6:1934185. doi: 10.3389/frhs.2026.1934185 (PMC13754886; doi:10.3389/frhs.2026.1934185)
Supplement: Supplementary file 1 [file Table1.docx]

Supplementary Material

# 1 Supplementary Tables

Table **S1**: Sensitivity analysis - number and proportion of persistent patients at day 1 and 180 in previous therapies by active ingredient/ATC classification **(**36 months retrospective observation period)

|  | **Patients with conventional preventive therapy, N (%)** | | | **Persistent patients with 180 days continuous prescription of the same preventive drug (incl. gap period), N (%)** | | |
| --- | --- | --- | --- | --- | --- | --- |
| **Preventive drug** | **Total* (N=144)** | **Female* (N=126)** | **Male* (N=18)** | **Total** | **Female** | **Male** |
| BoNTA (M03AX21) | 31 (22) | 26 (21) | 5 (28) | 31 (100) | 26 (100) | 5 (100) |
| Amitriptyline (N06AA09) | 85 (59) | 73 (58) | 12 (67) | 5 (4) | <5** | <5** |
| Flunarizine (N07CA03) | 58 (40) | 47 (37) | 11 (61) | 5 (4) | 5 (4) | 0 |
| Metoprolol (C07AB02) | 50 (35) | 41 (33) | 9 (50) | 0 | 0 | 0 |
| Propanolol (C07AA05) | 10 (7) | 10 (8) | 0 (0) | 0 | 0 | 0 |
| Topiramate (N03AX11, N02CX12) | 95 (66) | 85 (67,5) | 10 (56) | <5** | 0 | <5** |
| Valproate (N03AG01) | 5 (4) | <5** | <5** | 0 | 0 | 0 |

* Patients prescribed more than one preventive drug were counted multiple times
** Results based on 1-4 patients were masked for patient privacy
